# Biologic Augmentation Improves Rotator Cuff Integrity But Not Clinically Significant Patient Outcomes: A Meta-Analysis of Randomized Controlled Trials

**DOI:** 10.2106/JBJS.OA.26.00248

**Published:** 2026-09-22

**Authors:** Jake X Checketts, Stephen D Daniels, Corey J Schiffman, Frederick A Matsen, Jason E. Hsu

**Affiliations:** 1 Department of Orthopaedic Surgery and Sports Medicine, University of Washington Medical Center, Seattle, Washington

## Abstract

**Background:**

Biologic augmentation is increasingly used to enhance tendon healing after rotator cuff repair, with recent American Academy of Orthopaedic Surgeons guideline updates supporting certain implants. However, clinically significant benefits to the patient remain unclear. The primary objectives of this study were (1) to assess whether adding biologic augmentation to rotator cuff repair lowers retear rates and (2) to determine whether biologic augmentation yields superior patient-reported outcomes that exceed the minimal clinically important difference threshold for clinical significance.

**Methods:**

Systematic review and meta-analysis were carried out for Level I randomized controlled trials (RCTs) (PubMed, Embase, and CENTRAL) from inception to January 1, 2025, per Preferred Reporting Items for Systematic Reviews and Meta-Analyses guidelines. Only RCTs reporting postoperative imaging and patient-reported outcomes of American Shoulder & Elbow Surgeons (ASES), Constant, Simple Shoulder Test (SST), and University of California Los Angeles (UCLA) were included. Random-effects models evaluated retear rates and functional scores; subgroup analyses examined platelet rich plasma (PRP), patch, and other biologics. Mixed-effects models and correlations assessed the relationship between retear and function.

**Results:**

Twenty RCTs (1,327 shoulders) were included. Biologic augmentation reduced the retear rate by approximately 12% (14.8% vs. 26.7%; RR 0.62, 95% CI 0.50-0.76, p < 0.0001; I^2^ = 8%), with significant benefits for both PRP and patch augmentation. Intact repairs had clinically significantly higher ASES (+14.3, 95% CI 10.8-17.8) and Constant (+10.5, 95% CI 7.2-13.8) scores than retorn repairs. However, patient-reported outcomes were not clinically significantly better for augmented repairs (effect size ASES 0.9; Constant 2.18; SST 0.78; UCLA 1.01).

**Conclusion:**

In the included RCTs, biologic augmentation was associated with lower retear rates but not clinically significantly greater patient-reported outcomes after rotator cuff repair.

**Level of Evidence:**

Level I, evidence-based systematic review. See Instructions for Authors for a complete description of levels of evidence.

## Introduction

There have been significant advances in the management of rotator cuff tears. Despite advances in surgical techniques, avoidance of retear after repair is not guaranteed; structural failure rates remain considerable, especially in larger tears^1^. Such retears are associated with inferior patient outcomes and often necessitate revision procedures^1^. Surgeons have sought additional strategies to optimize healing and functional recovery. One promising approach is biologic augmentation of rotator cuff repairs, using adjunct therapies such as platelet-rich plasma (PRP) preparations, bioinductive collagen patches, acellular dermal allografts, mesenchymal stem cell–based treatments, and other biologic implants^1^.

This growing interest in biologic augmentation is reflected in recent research and in updated clinical guidelines. In the 2025 American Academy of Orthopaedic Surgeons (AAOS) Clinical Practice Guideline for Rotator Cuff Repair, there was a strong recommendation *against* routine use of platelet-derived products for augmentation, concluding that “biological augmentation of rotator cuff repair with platelet-derived products is not recommended for improving patient-reported outcomes; however, limited evidence supports the use of liquid PRP in the context of decreasing retear rates^2,3^.” More recently, the 2025 AAOS guideline update introduced a new strong recommendation in favor of augmenting repairs with a bioinductive collagen implant. It states that “the use of bioinductive tendon implants to augment rotator cuff repair or as an alternative to nonaugmented repair can lead to lower retear rates and better patient-reported outcomes^4^.” Notably, this recommendation was based on only 2 Level I studies available on bioinductive implants to date^4^. Evidence base for biologic augmentation remains limited, and consensus on its clinical efficacy has yet to be established.

Given the heightened interest in biologic augmentation and the still inconclusive evidence, we undertook a systematic review of the highest-level studies to clarify its benefits. In this review, we focused exclusively on Level I randomized controlled trials of rotator cuff repair with biologic augmentation. The primary objectives were twofold (1) to assess whether adding a biologic adjunct to rotator cuff repair improves tendon healing as reflected by lower retear rates and (2) to determine whether biologic augmentation yields superior patient-reported outcomes that exceed the minimal clinically important difference (MCID) threshold for clinical significance.

## Methods

### Search Strategy

We consulted the Preferred Reporting Items for Systematic Reviews and Meta-Analyses (PRISMA) 2020 checklist. We searched PubMed, Cochrane Central Register of Controlled Trials (CENTRAL), and Embase from inception through January 1, 2025. The full-search strategy is available in Appendix 1. Additional studies were identified through manual reference list screening and citation tracking.

### Study Selection

We screened studies by title and abstract, followed by full-text review. Senior authors were available to resolve concerns regarding inclusion via consensus or third-party adjudication. Eligible studies were randomized controlled trials with no more than 20% loss to follow-up published in English involving adults who underwent primary rotator cuff repair, with a minimum of 6 months clinical follow-up and postoperative imaging assessment of repair integrity with clinical outcomes reported. Exclusion criteria included revision rotator cuff repairs, pediatric populations, non-English language, animal/cadaveric models, partial-thickness nonsurgically treated tears or isolated subscapularis tears, and procedures involving tendon transfers, flaps, or arthroplasty. We did not screen for race and ethnicity.

### Data Extraction

We independently extracted study data, including author, publication year, level of evidence, sample size, patient demographics, surgical technique, augmentation type, tear size, fatty infiltration grade, imaging method, retear rates, patient-reported outcomes (e.g., ASES and Constant), complications, and follow-up duration. Where applicable, subgroup data (e.g., augmentation strategy) were extracted independently and analyzed separately. Age categories were recorded using midpoints. Tear sizes were standardized into the 4 categories (A-D) described by McElvaney et al^5^. Fatty infiltration was classified using the Goutallier system^6^ or Fuchs modification^7^, and the Global Fatty Degeneration Index (GFDI)^8^ was calculated as the mean of the grades for the supraspinatus, infraspinatus, and subscapularis. Augmentation strategies with more than 3 cohorts of similar method and/or product were placed into named groups for analysis, and augmentation strategies with less than 3 cohorts were labeled “other” for analysis.

Rotator cuff integrity was assessed using magnetic resonance imaging (MRI), ultrasound, or contrast CT (computed tomography). Most studies reported outcomes as either “intact” or “retorn.” For studies using the Sugaya^9^ classification, grades 1 to 3 were considered “intact” and grades 4 to 5 were categorized “retorn.”

### Risk of Bias Assessment

The Cochrane Risk of Bias 2.0 tool was used to assess methodological quality across 5 domains: (1) randomization process, (2) deviations from intended interventions, (3) missing outcome data, (4) measurement of the outcome, and (5) selection of the reported result. Overall risk was categorized low risk, some concerns, or high risk.

### Statistical Analysis

Study-level data on rotator cuff repair integrity and patient-reported outcomes were extracted. The retear rate was defined as imaging-confirmed retears divided by shoulders with postoperative imaging. Risk ratios (RRs) were calculated using the Mantel-Haenszel method and pooled with DerSimonian-Laird random-effects models. Mean differences (MDs) for PROMs (ASES, SST, and UCLA) were pooled using inverse-variance random-effects models. Heterogeneity was assessed with I^2^. Subgroup analyses by augmentation type were performed when sufficient data were available. Associations between retear rates, PROMs, and patient factors were evaluated using correlation, weighted regression, and mixed-effects models. Publication bias was assessed with funnel plots and Egger test. Analyses were 2-sided (p < 0.05).

## Results

### Search Results

A total of 2,571 studies were eligible after removal of duplicates. Following title and abstract screening, 586 full-text articles were reviewed. Of these, 530 were excluded. Fifty-four randomized controlled trials (RCTs) reported both rotator cuff integrity and clinical outcomes, but 3 were excluded due to excessive loss to follow-up or overlapping cohorts. Ultimately, 53 RCTs met all inclusion criteria, and of these, 20 included comparison arms between rotator cuffs receiving some type of biologic augmentation and those without such augmentation (Appendix Fig. 1).

### Study Characteristics

Study characteristics can be seen in Table I. The final analysis included 20 randomized controlled trials published between 2011 and 2024. A total of 1,327 shoulders were analyzed. Mean follow-up duration was 18.4 months (SD 9.2), and the mean follow-up rate across studies was 94.8%. The average patient age was 58.8 years, with 52% male participants and 21% active smokers across studies. Postoperative rotator cuff integrity was most frequently assessed using MRI (13 studies). Risk of bias was generally low, with 15 studies rated as low risk and 5 rated as having some concerns.

**TABLE I:** Study Characteristics of Included Randomized Controlled Trials

Author (Year)	Journal	Biologic Comparison	Cuff Integrity Assessment	Follow-Up (months)	Sample Size	Mean Age	Male (%)	Smokers (%)	Diabetic (%)	Risk of Bias
Randelli et al.^10^	*JSES*	PRP vs. no	MRI	24.0	45		46.7			Low risk
Ruiz-Moneo et al.^11^	*Arthroscopy*	PRP vs. no	Arthro-MRI	12.0	63	55.5	39.7	22.2	0.0	Low risk
D'Ambrosi et al.^12^	*Musculoskeletal Surgery*	PRP vs. no	Ultrasound	6.0	40		47.5			Some concerns
Bryant et al.^13^	*JSES*	Porcine vs. no porcine scaffold	MRA	24.0	58	56.6	87.9	15.5		Low risk
Chacon et al.^14^	*JSES*	Regeneten vs. suture repair	MRI	24.0	60		50.0	16.7	3.3	Low risk
Pandey et al.^15^	*JSES*	PRP vs. No PRP	US	24.0	102	54.3	72.5	0.0		Low risk
Liu et al.^16^	*OJSM*	PRP vs. PRP + booster vs. control	MRI	12.0	48	63.0	50.0			Low risk
Ruiz et al.^17^	*Arthroscopy*	Repair + regeneten vs. repair alone	MRI	12.0	120		50.8	25.0	12.5	Low risk
Lamas et al.^18^	*Trials*	MSC + collagen membrane vs. no MSC	MRI	12.0	13		61.5			Some concerns
Gumina et al.^19^	*JBJS*	platelet leukocyte membrane vs. none	MRI	12.0	76	61.0	53.9			Some concerns
Ebert et al.^20^	*AJSM*	PRP vs. no PRP	MRI	42.0	55		32.7			Some concerns
Oh et al.^21^	*Clinics in Orthopaedic Surgery*	HACMC vs. none	CT arthrography or US	12.0	80		48.8			Some concerns
Flury et al.^22^	*AJSM*	PRP vs. no PRP	MRI or US	24.0	103		36.9	28.2	0.0	Low risk
Kim et al.^23^	*AJSM*	Porcine atelocollagen vs. none	MRI	12.0	61		55.7	9.8	13.1	Low risk
Rossi et al.^24^	*AJSM*	Leukocyte poor PRP vs. control	MRI	12.0	96	56.1	56.2	19.8	8.3	Low risk
Jo et al.^25^	*AJSM*	PRP vs. no PRP	MRI or CTA	12.0	48		50.0			Low risk
Malavolta et al.^26^	*AJSM*	PRP vs. control	MRI	24.0	54		31.5	18.5	81.5	Low risk
Cole et al.^27^	*AJSM*	BMAC vs. control	MRI	36.5	82		69.5	34.1	7.3	Low risk
Zumstein et al.^28^	*JSES*	PRP fibrin clot vs. control	MRI	14.5	35	65.3	51.4	0.0		Low risk
Castricini et al.^29^	*AJSM*	PRP matrix vs. control	MRI	16.0	88		45.5			Low risk

BMAC = bone marrow aspirate concentrate, CT = computed tomography, CTA = CT arthrogram, MRI = magnetic resonance imaging, and PRP = platelet-rich plasma.

### Repair Integrity

**Fig. 1:**
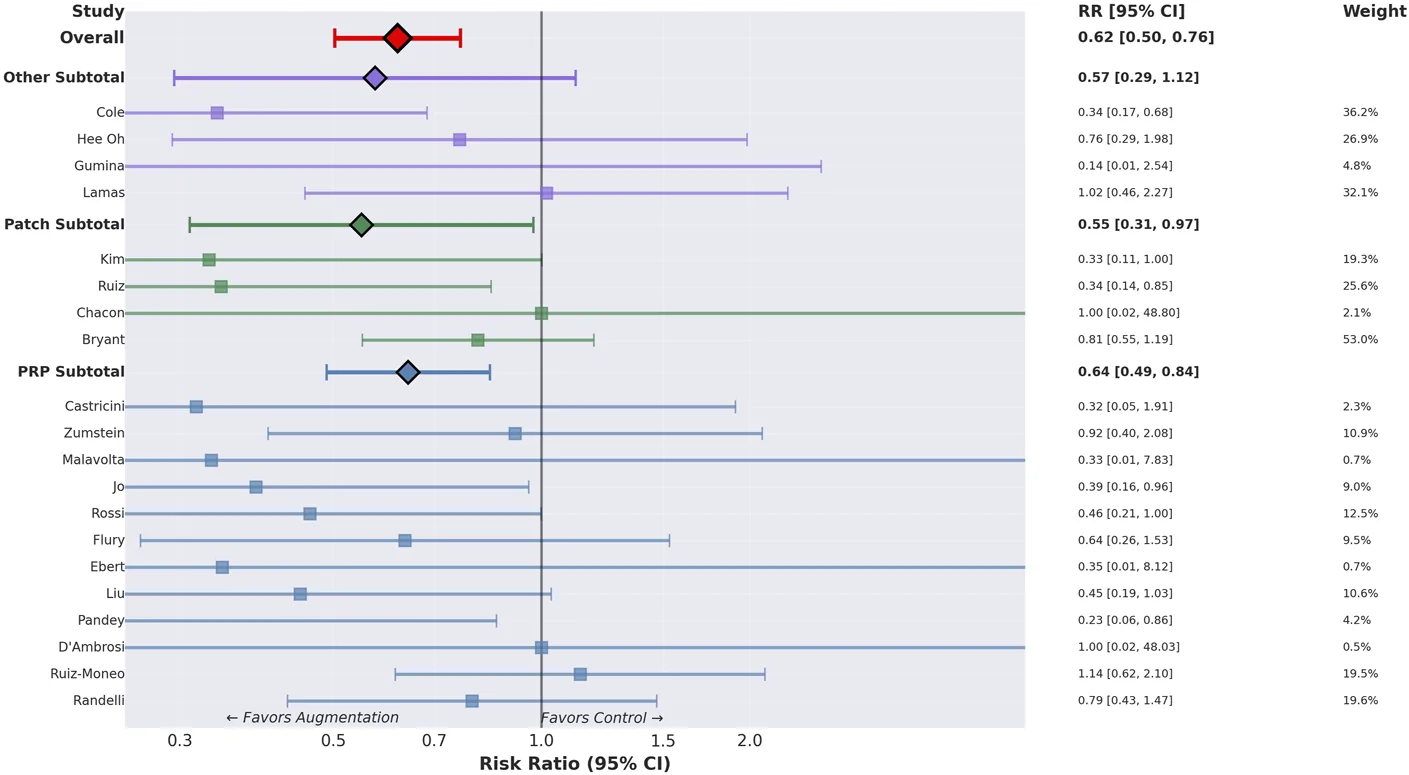
Retear rate-subgroup analysis by augmentation type. Forest plot displaying RRs with 95% CI for retear rates comparing biologic augmentation with control, stratified by augmentation type. The overall pooled effect (red diamond) demonstrates a significant reduction in retear risk with augmentation (RR = 0.62 [95% CI 0.50, 0.76]). Subgroup analysis reveals consistent benefit across all augmentation types: Patch augmentation (green; RR = 0.55 [0.31, 0.97]; 4 studies), PRP augmentation (blue; RR = 0.64 [0.49, 0.84]; 12 studies), and other biologics (purple; RR = 0.57 [0.29, 1.12]; 4 studies). Individual study estimates are shown as squares, with size proportional to study weight; subgroup and overall pooled estimates are shown as diamonds. The vertical line at RR = 1.0 represents no treatment effect. Values to the right of this line favor control; values to the left favor augmentation. Weights represent the relative contribution of each study to the pooled estimate. Analysis performed using a random-effects model with the DerSimonian-Laird method. CI = computed tomography, and RR = risk ratio.

The retear rate was 14.8% (98 of 662) in augmented repairs and 26.7% (173 of 648) in control repairs at a mean imaging follow-up of 15.0 months (Table II). Biologic augmentation was associated with a 38% relative risk reduction (risk ratio [RR] 0.62, 95% confidence interval [CI] 0.50-0.76, p < 0.0001; Fig. 1). Heterogeneity was I^2^ = 8%.

**TABLE II:** Retear Rate Comparisons Between Controls and Augmented Rotator Cuff Repairs

Group/Comparison	N (shoulders)	Retears (n, %)	Risk Ratio (95% CI)	p	I2 (%)
Control	648	173 (26.7)	—	—	—
All augmented vs. control	662	98 (14.8)	0.62 (0.50-0.76)	<0.0001	8
PRP vs. control	399	54 (13.5)	0.64 (0.49-0.84)	0.001	0
Patch vs. control	153	27 (17.6)	0.55 (0.31-0.97)	0.04	35
Other vs. control	110	17 (15.5)	0.57 (0.29-1.12)	0.08	44

CI = confidence interval, and PRP = platelet-rich plasma.

Subgroup difference: p = 0.048

Random-effects model (no continuity correction). Liu PRP arms separate. 20 RCTs, 40 arms, 1,310 shoulders.

Subgroup analysis showed significant reductions with platelet-rich plasma (RR 0.64, 95% CI 0.49-0.84, p = 0.001, I^2^ = 0%) and patch augmentation (RR 0.55, 95% CI 0.31-0.97, p = 0.04, I^2^ = 35%), but not other biologics (RR 0.57, p = 0.08). Subgroup difference was p = 0.048 (Fig. 1). Funnel plot and Egger test showed no publication bias (p = 0.69) (Appendix Fig. 2).

### Correlation of Repair Integrity and Patient-Reported Outcomes

**Fig. 2:**
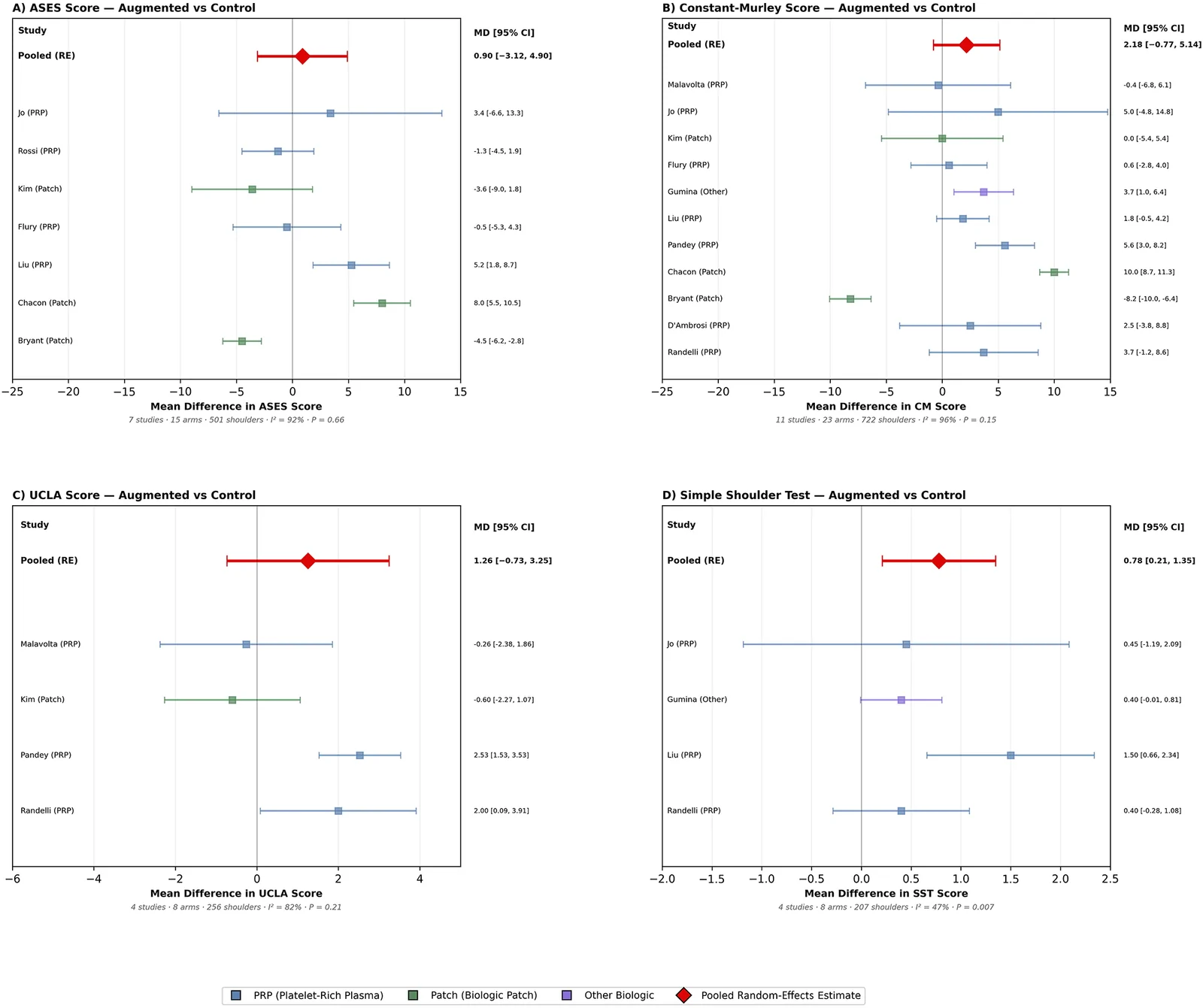
Forest plots comparing functional outcome scores between biologic augmentation and control groups in rotator cuff repair. (**Fig. 2-A**) ASEC score (7 studies, 501 shoulders), (**Fig. 2-B**) Constant-Murley score (11 studies, 722 shoulders), (**Fig. 2-C**) UCLA shoulder score (4 studies, 256 shoulders), and (**Fig. 2-D**) Simple Shoulder Test (4 studies, 207 shoulders). Squares represent individual study mean differences with 95% CIs; square color indicates treatment type (blue = PRP, green = patch, purple = other biologic). Red diamonds represent pooled random-effects estimates. Only the SST showed a statistically significant difference favoring augmentation (MD = 0.78; p = 0.007). ASES = American Shoulder and Elbow Surgeon, CI = confidence interval, and UCLA = University of California Los Angeles.

**Fig. 3:**
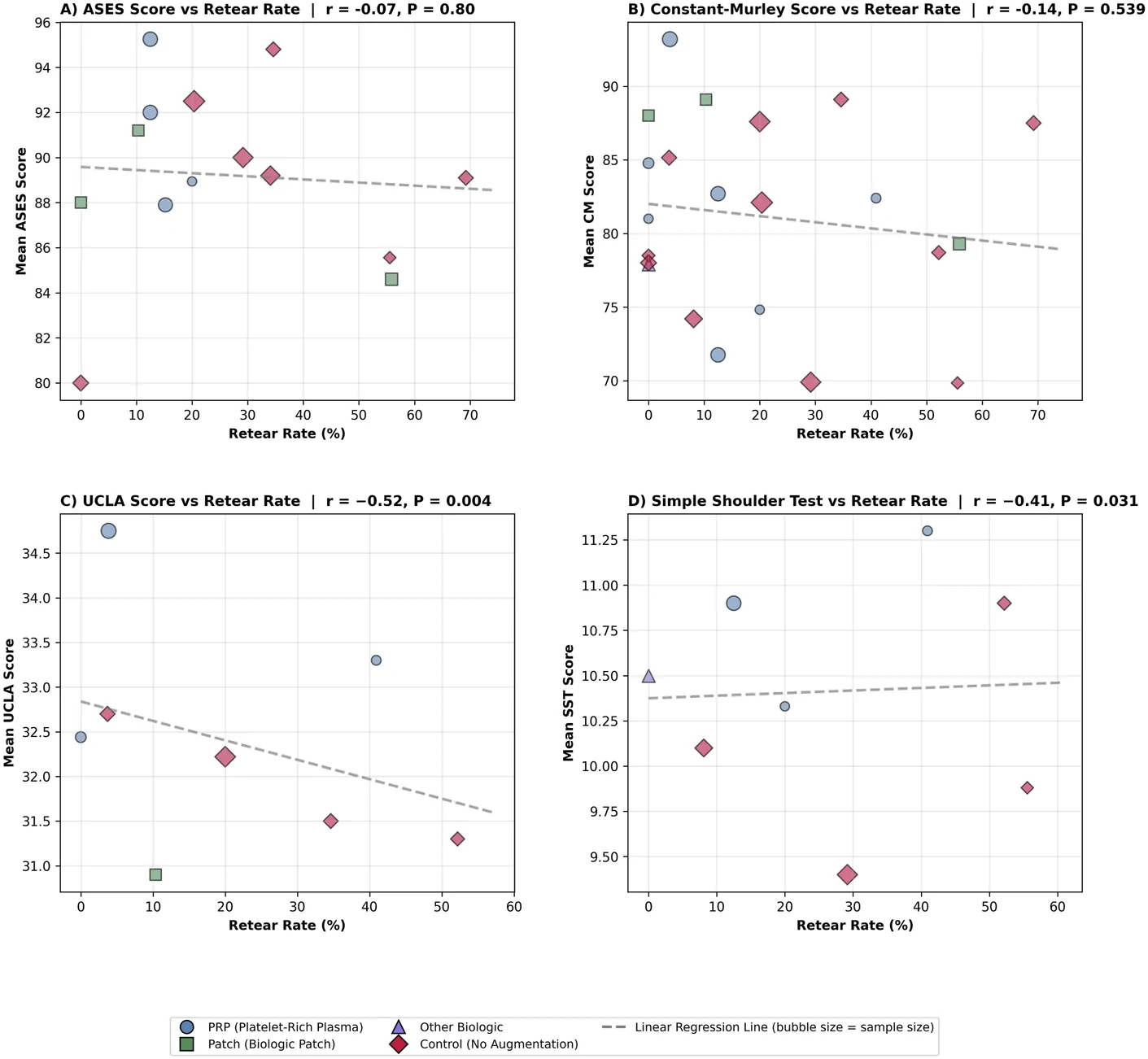
Relationship between retear rate and functional outcome scores by treatment type. **Fig. 3-A** ASES score, (**Fig. 3-B**) Constant-Murley score, (**Fig. 3-C**) UCLA score, and (**Fig. 3-D**) Simple Shoulder Test. Each data point represents a study arm, with bubble size proportional to sample size. Treatment groups are color-coded: blue circles = PRP, green squares = patch, purple triangles = other biologic, red diamonds = control. Dashed gray lines show overall regression trends. Significant negative correlations were observed for UCLA (r = −0.52; p = 0.004) and SST (r = −0.41; p = 0.031), indicating higher retear rates were associated with lower functional scores. ASES = American Shoulder and Elbow Surgeon, PRP = platelet rich plasma, SSt = Simple Shoulder Test, and UCLA = University of California Los Angeles.

Biologic augmentation produced improvements in postoperative patient-reported outcomes that did not reach clinical importance (Fig. 2). The pooled mean differences in final scores were 0.90 points for ASES (95% CI −3.12 to 4.90, p = 0.66; I^2^ = 92%; 7 studies, 15 arms, 501 shoulders), 2.18 points for the Constant-Murley score (95% CI −0.77 to 5.14, p = 0.15; I^2^ = 96%; 11 studies, 23 arms, 714 shoulders), 0.78 points for SST (95% CI 0.21-1.35, p = 0.007; I^2^ = 47%; 4 studies, 8 arms, 207 shoulders), and 1.26 points for UCLA (95% CI −0.73 to 3.25, p = 0.21; I^2^ = 82%; 4 studies, 8 arms, 256 shoulders). Patch augmentation significantly improved the Constant score by 3.85 points (95% CI 0.45-7.25, p = 0.027, I^2^ = 0%) but not ASES (−1.95 points, p = 0.33), SST (not available), or UCLA (−0.60 points, p = 0.60). PRP augmentation showed no significant benefit on any measure (all p > 0.16). Subgroup differences were not significant (all p > 0.25). The UCLA score demonstrated the strongest correlation with retear rate (r = −0.52, p = 0.004), followed by SST (r = −0.41, p = 0.031), Constant (r = −0.14, p = 0.54), and ASES (r = −0.07, p = 0.80). Similar patterns were observed for improvement scores (Fig. 3).

By contrast, when evaluating all patients irrespective of treatment type, postoperative tendon integrity was strongly associated with superior functional outcomes. Intact repairs outperformed retears by 14.3 points on ASES (95% CI 10.8-17.8, p < 0.0001; I^2^ = 45%), 10.5 points on Constant (95% CI 8.7-12.3, p < 0.0001; I^2^ = 42%), 1.6 points on SST (95% CI 1.1-2.1, p < 0.0001; I^2^ = 32%), and 3.3 points on UCLA (95% CI 1.2-5.4, p = 0.002; I^2^ = 68%).

Linear mixed-effects models (study as random intercept) confirmed retear rate as the dominant driver of outcome. Each 10% increase in retear rate was associated with reductions of 4.8 points in Constant (95% CI −5.9 to −3.7, p < 0.001), 1.8 points in SST (95% CI −2.5 to −1.1, p < 0.001), and 3.8 points in UCLA (95% CI −6.1 to −1.5, p = 0.001), with no significant effect on ASES. After adjustment for retear rate, augmentation type was nonsignificant in all models (Appendix Fig. 3).

Sensitivity analyses (separating multiarm studies, excluding small studies or outliers) and publication bias assessment (symmetrical funnel plots, Egger p > 0.71 for all outcomes) did not alter the finding (Appendix Fig. 4).

## Discussion

Biologic augmentation of rotator cuff repair significantly reduced the postoperative retear rate by 12% across 20 randomized trials, with benefits from platelet-rich plasma (PRP) and patch-based strategies. No patient-reported outcome showed clinically meaningful improvement with biologic augmentation, all below established MCID thresholds^30-33^ and nonsignificant except SST (p = 0.007). These data suggest that more selective use of biologic augmentation may be necessary.

With a control healing rate of 73.3%, augmentation prevents retear in only ∼12% of shoulders (absolute risk reduction 11.9%). The large functional advantage of an intact repair (10.5-14.3 points on ASES/Constant) is therefore diluted across the entire cohort, yielding an expected population-level gain of only ∼1.3 to 1.7 points. This pattern aligns with prior meta-analyses^34-36^ but is particularly striking given our focus on Level I evidence.

Recent AAOS guideline updates highlight the tension between structural and functional evidence. The 2025 Clinical Practice Guideline^4^ now provides a strong recommendation for bioinductive implants to reduce retears and improve outcomes, upgrading dermal allografts to moderate and limiting PRP to select cases—shifts from the 2019 guideline's caution against routine biologics. These changes reflect emerging Level I data on healing but overlook the functional disconnect our meta-analysis reveals. While patches show the strongest retear reduction (RR 0.55, p = 0.04), the lack of MCID-exceeding PROM gains raises questions about cost-effectiveness, with bioinductive implants costing $5,000–$10,000 per case^37,38^. Financial conflicts could potentially influence study design, outcome selection, or interpretation. Owing to the strong recommendations within AAOS guidelines, surgeons may face medicolegal risks if retears occur without augmentation, potentially shifting standard of care despite uncertain patient benefits. Cost-utility analyses and long-term (>2 years) RCTs are essential to balance healing gains against functional, economic, and conflict-related realities.

Beyond the retear rate, better metrics for clinical success may include composite endpoints such as “MCID achievement + intact repair” or patient-specific thresholds (e.g., return to work/sport). Fatty infiltration (FDI) and retraction, balanced in our cohorts (p > 0.05), warrant subgroup exploration in future trials to identify responders. Machine learning models integrating imaging, PROMs, and biologics could predict individualized outcomes, moving beyond binary retear to quality-of-healing metrics.

Strengths of this review include exclusive focus on Level I RCTs, comprehensive imaging-confirmed retear data (1,310 shoulders), proper handling of shared controls, and novel PROM sensitivity comparisons. Limitations encompass potential publication bias (though Egger p > 0.69), variable follow-up (mean 15 months), exclusion of race and ethnicity screening, and exclusion of non-English studies. In addition, it is a limitation of our study that some of the included studies used ultrasound rather than MRI to evaluate cuff integrity. Heterogeneity in PROMs (I^2^ up to 92%) reflects real-world variability in tear characteristics and repair techniques, and we did not differentiate between the degree of fatty infiltration and tear size in our analysis, which limits our findings. Furthermore, PRP delivery and methodology was not the same between studies, and several different patch options were used, but in our analysis, we simply included them in the “PRP” or “patch” cohort. Furthermore, some patient-reported outcome measures were reported less frequently among our sample than others, limiting the associations and findings regarding these measures.

In conclusion, biologic augmentation strategies lead to a lower retear rate in the published literature, but clinically significant functional benefit is not seen in the populations studied in the existing RCTs. Current biologic augmentation strategies heal too few additional patients to produce perceptible improvement in typical tear populations. Surgeons may consider reserving these costly adjuncts for massive or high-risk tears with poor intrinsic healing potential and await evidence that functional gains justify both expense and evolving guideline recommendations.

## Funding

No outside funding was received for conducting this study.

## Appendix

Supporting material provided by the authors is posted with the online version of this article as a data supplement at jbjs.org (https://links.lww.com/JBJSOA/B346, https://links.lww.com/JBJSOA/B348, https://links.lww.com/JBJSOA/B349, https://links.lww.com/JBJSOA/B350, https://links.lww.com/JBJSOA/B351). This content was not copyedited or verified by JBJS.
